# Evaluation of Scat Deposition Transects versus Radio Telemetry for Developing a Species Distribution Model for a Rare Desert Carnivore, the Kit Fox

**DOI:** 10.1371/journal.pone.0138995

**Published:** 2015-10-14

**Authors:** Steven J. Dempsey, Eric M. Gese, Bryan M. Kluever, Robert C. Lonsinger, Lisette P. Waits

**Affiliations:** 1 Department of Wildland Resources, Utah State University, Logan, Utah, United States of America; 2 United States Department of Agriculture, Wildlife Services, National Wildlife Research Center, Department of Wildland Resources, Utah State University, Logan, Utah, United States of America; 3 Department of Fish and Wildlife Sciences, University of Idaho, Moscow, Idaho, United States of America; Smithsonian Conservation Biology Institute, UNITED STATES

## Abstract

Development and evaluation of noninvasive methods for monitoring species distribution and abundance is a growing area of ecological research. While noninvasive methods have the advantage of reduced risk of negative factors associated with capture, comparisons to methods using more traditional invasive sampling is lacking. Historically kit foxes (*Vulpes macrotis*) occupied the desert and semi-arid regions of southwestern North America. Once the most abundant carnivore in the Great Basin Desert of Utah, the species is now considered rare. In recent decades, attempts have been made to model the environmental variables influencing kit fox distribution. Using noninvasive scat deposition surveys for determination of kit fox presence, we modeled resource selection functions to predict kit fox distribution using three popular techniques (Maxent, fixed-effects, and mixed-effects generalized linear models) and compared these with similar models developed from invasive sampling (telemetry locations from radio-collared foxes). Resource selection functions were developed using a combination of landscape variables including elevation, slope, aspect, vegetation height, and soil type. All models were tested against subsequent scat collections as a method of model validation. We demonstrate the importance of comparing multiple model types for development of resource selection functions used to predict a species distribution, and evaluating the importance of environmental variables on species distribution. All models we examined showed a large effect of elevation on kit fox presence, followed by slope and vegetation height. However, the invasive sampling method (i.e., radio-telemetry) appeared to be better at determining resource selection, and therefore may be more robust in predicting kit fox distribution. In contrast, the distribution maps created from the noninvasive sampling (i.e., scat transects) were significantly different than the invasive method, thus scat transects may be appropriate when used in an occupancy framework to predict species distribution. We concluded that while scat deposition transects may be useful for monitoring kit fox abundance and possibly occupancy, they do not appear to be appropriate for determining resource selection. On our study area, scat transects were biased to roadways, while data collected using radio-telemetry was dictated by movements of the kit foxes themselves. We recommend that future studies applying noninvasive scat sampling should consider a more robust random sampling design across the landscape (e.g., random transects or more complete road coverage) that would then provide a more accurate and unbiased depiction of resource selection useful to predict kit fox distribution.

## Introduction

Many carnivore populations are declining worldwide [1, 2]. Determining the distribution and abundance of rare carnivores is important for developing informed conservation and management plans [3]. Difficulties inherent in monitoring carnivores is their tendency to be elusive, wary, far-ranging, occupy remote areas or densely vegetated habitats, or exist at low density, making determination of their distribution and abundance a daunting task for ecologists and wildlife management agencies [4, 5]. Oftentimes invasive sampling is employed to monitor carnivore abundance using capture-mark-recapture, radio-collaring, or catch-per-unit-effort [4]. These methods are often costly, stressful and risky to the animal, and time consuming [6]. The use of noninvasive sampling [5, 7] has the advantage of not needing to capture the animal and is generally less expensive. However, the relationship between an indirect noninvasive method and a species abundance and distribution should be evaluated [4].

Historically, kit foxes (*Vulpes macrotis*) occupied the desert and semi-arid regions of southwestern North America ranging from Idaho to central Mexico [8]. Their range-wide decline had warranted the kit fox to be listed as endangered in Colorado, threatened in California and Oregon, and designated as a state sensitive species in Idaho and Utah [9]. Although listed and protected in several states, a comprehensive study of kit fox distribution is lacking, with the majority of studies focused on the endangered subspecies, the San Joaquin kit fox (*V*. *macrotis mutica*), leaving a need for greater knowledge of monitoring methods for the species across its entire range.

Once considered the most abundant carnivore in western Utah [10, 11], the kit fox has been in decline over the past three decades [12–14]. Previous studies provide evidence of environmental variables affecting kit fox spatial distribution. In early reports on the kit fox population in western Utah [11], kit foxes were described as using areas that were predominately flat and featureless with sparse vegetation. They selected areas with silt and clay soils or sandy dunes and their den sites were mostly found in greasewood. In a later study on the same geographic area [15], kit fox dens were now found in grasslands with low vegetation. In western Colorado, kit foxes existed at elevations between 1,463 to 1,829 m and in areas with high clay to clay-loam soils [16]. Vegetation height was low at den sites and sparsely distributed [16]. In addition, they found most den entrances had a southerly aspect. Additional studies found kit foxes to be present in areas with low ground cover, loamy desert soils, at elevations <1,675 m, and vegetation height at den sites was generally >22 cm [8].

In the past few decades, attempts have been made to model the environmental variables influencing kit fox space use. Researchers reported the geographic range of kit foxes was closely associated with open flat habitats with little vegetative cover and kit foxes spent more time in greasewood flats than riparian areas [17]. Other researchers modeled kit fox occurrence using capture rates, and used a standardized regression approach to evaluate the influence of land development, ruggedness, fenced lands, and burned areas [18]. They also used linear regression to compare space use of foxes with coyote and lagomorph abundance, but found no significant relationship. They determined that topographic ruggedness was the only consistent variable affecting the spatial distribution of kit foxes. An early attempt at GIS modeling of habitat use by kit foxes based on land-cover type and roads found grasslands to be important to kit foxes [19].

Kit fox distribution has not been modeled using more recently developed techniques. One such technique is using resource selection functions to predict species distribution using presence/availability data [20, 21]. These species distribution models (SDMs) can vary greatly depending on which techniques are used to build the model, but usually require ‘use’ or ‘presence’ data [20, 21]. Such data is traditionally obtained through animal locations via radio telemetry or global positioning system collars; considered invasive sampling since the animal must be captured. But increasingly, noninvasive techniques have been employed to detect species presence [4–5, 22].

Carnivores are generally difficult to survey because they exist at low densities, are usually nocturnal and elusive, as well as wary of humans [4, 12, 23–25]. The kit fox population in the western desert of Utah is considered declining in abundance, of low density, and widely dispersed [13, 26, 27]. Given these conditions, obtaining presence data from traditional invasive sampling (i.e., capture, radio-collar, relocate) may be difficult and there is a need for evaluating a passive noninvasive technique to determine species’ presence [4, 7].

In this study we modeled kit fox distributions using two generalized linear model types and a Maxent approach for creating resource selection functions. We used scat deposition surveys as a noninvasive sampling technique to determine animal presence, and then created the SDMs using environmental variables related to topography, soil, and vegetation. We also developed similar SDMs using invasive sampling (i.e., capture, radio-collar, and relocate) data from radio-collared foxes. We then examined the relative probability of occurrence or resource selection between the noninvasive technique and invasive techniques. Our objectives were to 1) create resource selection functions used to predict species distribution models using scat transect surveys and radio-telemetry locations, 2) evaluate the performance of the species distribution models derived from the noninvasive and invasive techniques; 3) validate the species distribution models using scat transect data independent from model creation; and 4) provide recommendations on the use of invasive versus noninvasive sampling for creating species distribution models for a rare desert carnivore, the kit fox.

## Materials and Methods

### Ethics Statement

Fieldwork was approved and sanctioned by the United States Department of Agriculture’s National Wildlife Research Center and the United States Army’s Dugway Proving Ground. Permission to access land on the Dugway Proving Ground was obtained from the United States Army; permission to access Bureau of Land Management property was obtained from the Bureau of Land Management.

Capture and handling protocols were reviewed and approved by the Institutional Animal Care and Use Committees (IACUC) at the United States Department of Agriculture’s National Wildlife Research Center (QA-1734) and Utah State University (#1438). Permits to capture, handle, and radio-collar kit foxes were obtained from the Utah Division of Wildlife Resources (COR #4COLL8322). All relevant data are within the paper and its Supporting Information files.

### Study Area

We conducted our research on 879 km^2^ of the Dugway Proving Ground and the adjoining land managed by the Bureau of Land Management, located approximately 128 km southwest of Salt Lake City, in Tooele County, Utah. Elevations ranged from 1302 m to 2137 m. The study site was in the Great Basin Desert and was characterized as a cold desert. Winters were cold, while summers were hot and dry, with the majority of precipitation occurring in the spring. Average maximum temperatures range from 3.3°C in January to 34.7°C in July [13]. Average minimum temperatures range from -8.8°C in January to 16.3°C in July [13]. Mean annual precipitation is 20.07 cm [13]. The study area consisted of predominately flat playa punctuated with steep mountain ranges. The lowest areas consisted of salt playa flats sparsely vegetated with pickleweed (*Allenrolfea occidentalis*). At areas of slightly higher elevations, soils were less salty and supported a cold desert chenopod shrub community consisting predominately of shadscale (*Atriplex confertifolia*) and gray molly (*Kochia americana*). At similar elevations, greasewood (*Sarcobatus vermiculatus*) communities would be found with mound saltbrush (*Atriplex gardneri*) and Torrey seepweed (*Suaeda torreyana*). Higher elevations consisted of vegetated sand dunes including fourwing saltbush (*Atriplex canescens*), greasewood, rabbitbrush (*Chrysothamnus* spp.), shadscale, and horsebrush (*Tetradymia glabrata*). Near the bases of the higher steep mountains were shrubsteppe communities of sagebrush (*Artemisia* spp.), rabbitbrush, Nevada ephedra (*Ephedra nevadensis*), greasewood, and shadscale. At the highest elevation was a Utah juniper (*Juniperus osteosperma*) community including black sagebrush (*Artemisia nova*) and bluebunch wheatgrass (*Elymus spicatus*). Where wildfires had occurred along the foothills, cheatgrass (*Bromus tectorum*), tall tumble mustard (*Sisymbrium altissimum*), and Russian thistle (*Salsola kali*) was common within communities of sagebrush, rabbitbrush, and juniper [13].

### Noninvasive Sampling

**Fig 1:**
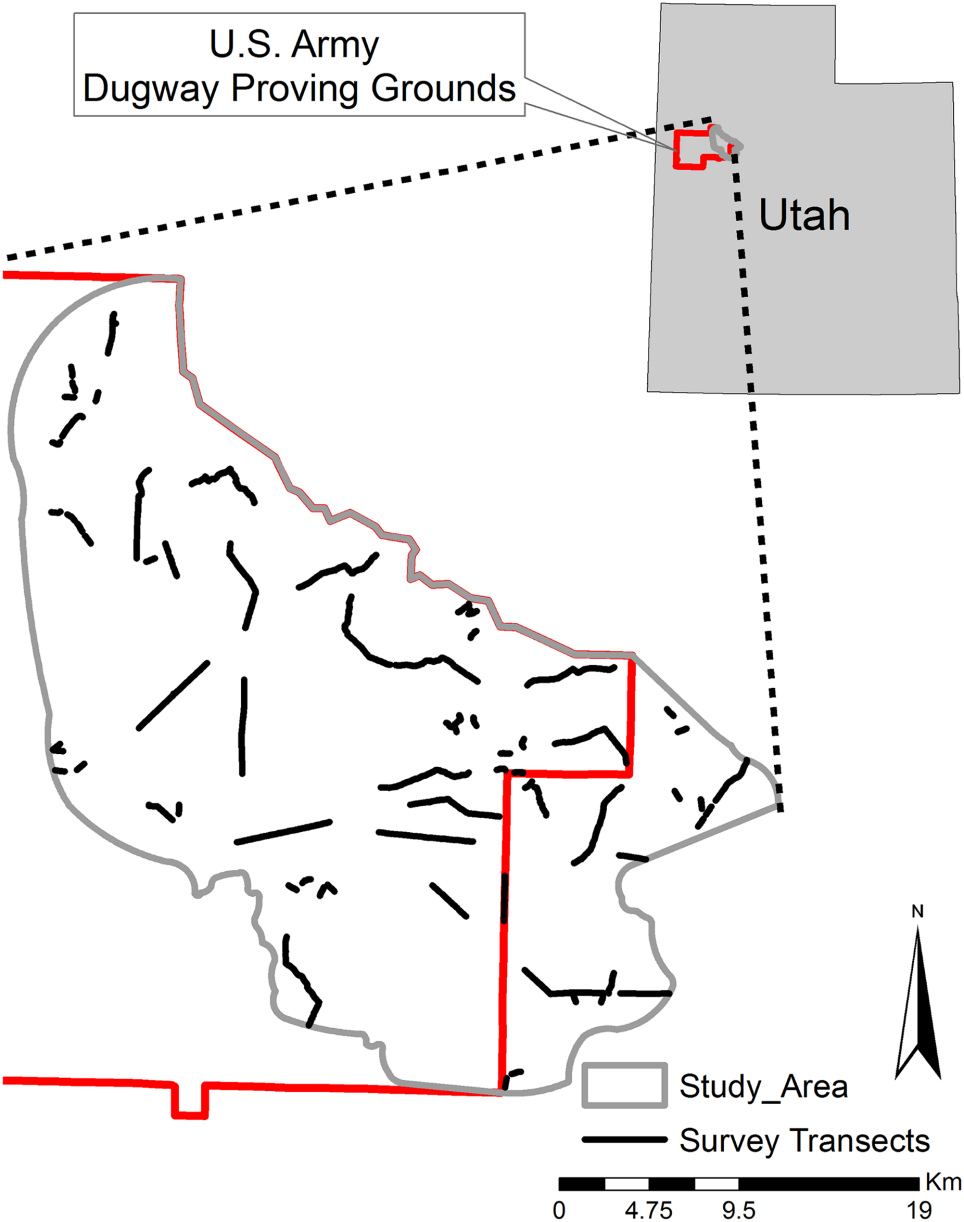
Study area and survey transect layout at Dugway Proving Ground, Utah.

In order to estimate kit fox distribution in the study area using a noninvasive sampling technique, we conducted scat deposition surveys [28, 29] along 15 5-km (Fig 1) transects distributed randomly along available roads with the constraint of being as linear as possible and having year-round access (limitations included military closures and low-lying seasonally inundated greasewood areas). During the breeding season of 2013, we added an additional five 5-km transects and 69 0.5-km transects randomly placed within the study area to provide a wider distribution of transects across the study area for another study examining the use of noninvasive genetic sampling as a monitoring technique for determining population demographics of kit foxes [30, 31]. We initially walked the transect to clear any scat from the road surface, then returned approximately 14 days later to walk and count the number of scats deposited [28, 29]. Following recommendations [28, 29], we walked each transect in both directions to reduce missing scats. We recorded scat location and type (species) on a handheld GPS unit. Kit fox scats were distinguished from other carnivore scats (mainly coyote) by size, shape, and odor [27, 32, 33].

We conducted scat deposition surveys during each of the three biological kit fox seasons (breeding, pup-rearing, and dispersal) during December 2009 through March 2013. We defined biological seasons by behavior and energetic needs of kit foxes [11, 15, 34]: breeding (15 December– 14 April), pup-rearing (15 April– 14 August) and dispersal (15 August– 14 December). Collected scats were then separated into training and testing datasets. The training dataset consisted of all scats collected during the first 2 years of sampling; the testing dataset consisted of the scats collected during the third and final year of sampling.

### Invasive Sampling

In order to determine kit fox distribution in the study area using an invasive sampling technique, we captured kit foxes via transect trapping and opportunistically at known den sites, using box traps (Tomahawk Live Trap LLC, Hazelhurst, Wisconsin). Traps were deployed in the evening and checked early morning each day. We coaxed captured foxes into a canvas bag placed at the edge of the trap, then restrained by personnel wearing thick leather gloves [15]. Foxes were weighed, sexed, ear tagged, and fitted with a 30–50 g radio-collar (Advanced Telemetry Systems, Isanti, Minnesota). The radio-collars included a mortality sensor that activated after 4 hours of non-motion and weighed <5% of body mass [29, 34, 35]. We handled all foxes without using immobilizing drugs and released them at the capture site.

We collected animal locations ≥3 times per week using a portable receiver (Communications Specialists, Inc., Orange, California) and a handheld Yagi antenna. We triangulated an animal’s location using ≥2 compass bearings, each >20° but <160° apart, for each animal within 20 minutes [13, 26]. Positions were then calculated using program Locate III (Pacer Computing, Tatamagouche, Nova Scotia). For each week, we temporally distributed telemetry sampling by collecting two crepuscular (hunting) locations via triangulation, and one den (resting) location by homing in on the animal during daylight hours. To reduce auto-correlation and retain temporal independence between locations each crepuscular sample was separated by >12 hours and a difference of >2 hours in the time of day of each location [36–38].

### Environmental Variables

All environmental (elevation, slope, aspect, soil type) data were available from GIS databases (Utah Automated Geographic Reference Center; http://gis.utah.gov). Soils in the study area were classified into 4 major classes: silt (SLT), fine sand (FS), blocky loam (BL_L), and gravel (GRV) (see Table 1 for detailed descriptions). A vegetation height layer was downloaded from the Landfire database (http://landfire.cr.usgs.gov/). This layer was generated separately for tree, shrub, and herbaceous cover life forms and determined by the average height (m) weighted by species cover based off of existing vegetation type. All layers were re-projected to 30 m x 30 m cells (the largest cell extent of any layer) and confined to the same spatial extent and distribution as the study area to ensure layer compatibility. After reprojection, each layer consisted of 977,122 cells. All spatial processing was completed using ArcGIS 10 (ESRI, Redlands, California).

**Table 1:** Soil reclassification based on major formation type adapted from the Utah Automated Geographic Reference Center (http://gis.utah.gov).

Reclassification	Silt (SLT)	Fine Sand (FS)	Blocky_Loam (BL_L)	Gravel (GRV)
Soil Types	Playa Loam	Yenrab fine sand	Kapod stony loam	Hiko Peak gravelly loam
	Taylorsflat Loam	Medburn fine sandy loam	Amtof rock outcrop complex	Hiko Peak-Checkett complex
	Pits	Tooele fine sandy loam	Checkett rock outcrop complex	Hiko Peak-Taylorsflat complex
	Saltair-Playas complex	Berent-Hiko Peak complex	Kapod very cobbly loam	Cliffdown gravelly sandy loam
	Skumpah-Yenrab complex	Yenrab-Tooele complex	Reywatt broad rock outcrop	Izamatch-Cliffdown complex
	Skumpah silt loam		Dune land	
	Timpie silt Loam		Hiko Peak very stony loam	

### Model Construction

Following a use–available design, we developed fixed-effects generalized linear models (GLM) and mixed-effects generalized linear models (GLMM) with a logistic function using the lme4 (Version 0.999375–37) package in R (version 2.12.1 [39]). Two sets of SDMs were built for comparison of noninvasive and invasive surveying to obtain “use” data. Set 1: noninvasive surveying, included all scats collected during the training sampling for use data. Set 2: invasive surveying, included all telemetry locations collected for used data. “Available” locations were created using 10,000 random locations [40], from within the study area and were the same number of locations used in the Maxent model (described below). We considered all biologically relevant (based on previously described studies) combinations of the environmental layers [41]. Elevation, slope, aspect, and vegetation height were continuous variables and soil type was the only categorical variable. To limit the number of parameters and improve model fit, we reclassified soil type into four broad classes, as previously described. In the GLMM, the survey transect was assigned as a random intercept to account for the possible correlation among scats found along different types of transects, to take into account the effects of road type (i.e., dirt two-track roads versus gravel roads) and road substrate that could influence differences in scat detection [42, 43]. For the telemetry data set, the individual kit fox was assigned as the random intercept to account for possible individual variation in space use. All variables included in any model had low Pearson correlations (<0.28). Models were ranked by Akaike information criterion corrected for small sample sizes (AICc) [21, 41, 44, 45]. The *β* coefficient values were calculated from a model average of all reasonable models, <2 ΔAICc.

We also created species distribution models from the noninvasive and invasive sampling datasets using a Maximum Entropy Model (Maxent). As the predictor in the Maxent models we used the scat locations from the training dataset and the radio-telemetry locations. The same environmental variables (elevation, slope, aspect, soil, vegetation height) were included in Maxent modeling [46]. Using default settings, Maxent creates 10,000 random “available” locations (see [47] for a detailed explanation of the Maxent modeling process). We kept the default tuning parameters in Maxent [47–49] and selected a random seed for 100 replicates with cross-validation.

### Model Evaluation

To test the performance of the models, two techniques were examined: AICc and area under the receiver operating curve (AUC). AICc scores were calculated for both GLM and GLMMs. The use of AICc has been shown appropriate for generalized linear models [21, 41, 45] and is generally considered a good test of model fit. Recently, researchers demonstrated AICc does not improve model evaluation in Maxent and suggested it should not be used [49], therefore, for the Maxent models, AUC scores were evaluated. Similar to the Wilcoxon test of ranks, AUC can be interpreted as a probability of a correct classification or prediction [50, 51]. Using AUC has generally been found to be the best evaluator of model performance [52] and considered highly effective [53], while not being dependent on thresholds [53, 54]. We followed the general classifications of ranking model accuracy in AUC scores; 0.5–0.7: low accuracy, 0.7–0.9: useful application, and >0.9: high accuracy [21, 55, 56]. The fixed-effects model AUC score was calculated using the ROCR function (ROCR Package Version 1.0–4) in R [39]. Maxent provides the AUC scores by default, using all test locations and the 10,000 random samples (see [57] for detailed explanation). The random term in the mixed effects GLM precludes the use of AUC to evaluate model performance.

### Model Validation

The model averaged *β* coefficient values from all reasonable models (<2 ΔAICc) were used to create species distribution models (SDMs) in ArcGIS 10 [57]. The SDMs were created at the same scale as the largest environmental layer (30 x 30 m cells) used to construct the models. Then the models were assessed using test scat dataset withheld from the training dataset [54, 58, 59]. As a test of model validity, we overlaid the test scats on the SDMs and determined if they were located in areas as predicted by the model to be where kit fox detections should occur. The habitat values predicted by each model were grouped into 10 bins using the quantile method in ArcGIS 10 which divided the bins into equal sizes by the number of pixels. The number of test scats that fell into each bin class was then counted, and the frequency of the test scats for the three noninvasive distribution models were compared to the same invasive distribution models. This allowed us to compare the frequency of test scats that fell into each bin class while controlling for bins to be of equal size.

## Results

### Sampling

We conducted noninvasive scat deposition surveys from December 2009 through March 2013. Sampling effort across biological seasons was similar between noninvasive and invasive sampling techniques. The training dataset consisted of 226 scats found along the transects. The subsequent test dataset consisted of 90 scats collected during the third year of sampling along the transects and covered all three biological seasons. During our invasive sampling, from December 2009 to April 2012, we accumulated 6,221 trap nights and captured 45 (26 females, 19 males) foxes across the study area. During the study we obtained 4,498 fox telemetry locations: 1,487 in the breeding, 1,464 in the dispersal, and 1,547 locations during the pup-rearing season.

### Models

There was considerable variation across all model types likely as a reflection of the two different data types (scat locations versus telemetry locations). All possible combinations of scat GLMs produced 7 models that fit our criteria of <2 ΔAICc. The top-ranked model included elevation, slope, vegetation height, and blocky-loam soils (use = β_0_ + β_1_Elevation + β_2_Slope + β_3_Height + β_4_BL_L; AICc = 1961.43). The telemetry GLMs produced 5 models that fit our criteria of <2 ΔAICc. The top-ranked model included elevation, slope, and all soil categories except for the finest silts (use = β_0_ + β_1_Elevation + β_2_Slope + β_3_BL_L+ β_4_FS+ β_5_GRV; AICc = 16250.46). The scat GLMM produced 15 models that fit our criteria. The top-ranked model included elevation and the two soil categories related to larger soil types (use = β_0_ + β_1_Elevation + β_2_BL_L+ β_3_GRV; AICc = 330.45). The telemetry GLMM produced 11 models that fit our criteria. The top-ranked model included slope and three soil classes (use = β_0_ + β_1_Slope + β_2_BL_L+ β_3_FS+ β_4_GRV; AICc = 10518.45). All *β* coefficients are reported for the GLMs and GLMMs for the noninvasive and invasive sampling (Table 2).

**Table 2:** Model averaged *β* coefficients, standard errors (SE), model weights, and P values of covariates for the GLM and GLMM for scat transects and telemetry locations, Dugway Proving Ground, Utah.

Model	Covariate	*β Coefficient*	*SE*	*Weight*	*P*
**Scat GLM**	Aspect	0.0002	0.0004	0.3179	0.6397
	Elevation	-0.0030	0.0025	0.9584	0.2215
	Slope	-0.4368	0.3184	1.0000	0.1715
	Height	0.0012	0.0009	1.0000	0.1671
	BL_L	-2.2915	1.8908	0.9968	0.2268
	FS				
	GRV	-0.0484	0.1475	0.3147	0.7434
	SLT				
**Telemetry GLM**	Aspect	-0.0002	0.0001	1.0000	0.2027
	Elevation	-0.0003	0.0002	1.0000	0.2026
	Slope	-0.0165	0.0128	1.0000	0.1998
	Height	0.0001	0.0001	1.0000	0.1987
	BL_L	0.4548	0.3629	0.7655	0.2101
	FS	-0.0846	0.1056	0.7036	0.4234
	GRV	-0.3740	0.3036	0.7655	0.2181
	SLT	0.0153	0.0790	0.7036	0.8465
**Scat GLMM**	Aspect	0.0002	0.0006	0.1518	0.6811
	Elevation	0.0098	0.0098	1.0000	0.3140
	Slope	-0.1501	0.1897	0.4851	0.4296
	Height				
	BL_L	-3.2165	3.5713	0.8050	0.3687
	FS	0.3107	3.7931	0.1476	0.9348
	GRV	-0.5223	0.4976	0.5937	0.2950
	SLT	0.3017	0.8619	0.1476	0.7266
**Telemetry GLMM**	Aspect	-0.0001	0.0001	1.0000	0.2872
	Elevation	-0.0001	0.0001	0.5029	0.4386
	Slope	-0.0064	0.0059	1.0000	0.2829
	Height				
	BL_L	0.5780	0.5370	0.7949	0.2818
	FS	-0.1512	0.6076	0.6208	0.8035
	GRV	-0.3287	0.1534	0.6188	0.0321
	SLT	0.0909	0.3585	0.7876	0.7998

Based upon AUC values, the Maxent model constructed from scat locations had the best performance of the AUC-ranked models developed (AUC = 0.832). The AUC was calculated for the top-ranked fixed-effects model (AUC = 0.755) and was slightly less than the Maxent model. The telemetry model (AUC = 0.713) had a lower fit than both the Maxent and fixed-effects models. All three models, the Maxent, fixed-effects, and telemetry models all fell within the criteria (0.7–0.9) of having useful application [50, 51]. All these models showed the most important variables to be elevation, slope, height, and BL_L soil type. The models showed the most important variables to be elevation and slope, followed by vegetation height, soil type, and aspect. In the scat Maxent model, elevation had 45% of the model contribution followed by slope, height, and soil type with similar contributions of 18%, 17%, and 15%, respectively; aspect had a very small contribution of 5%. In the telemetry Maxent model, elevation’s contribution increased to 51%. The contribution of slope greatly increased from 18% in the scat model to 44% using the telemetry locations. Aspect’s contribution at 4% was similar to the scat model; while soil and height had minimal contribution with both <1%.

### Model Validation and Evaluation

**Fig 2:**
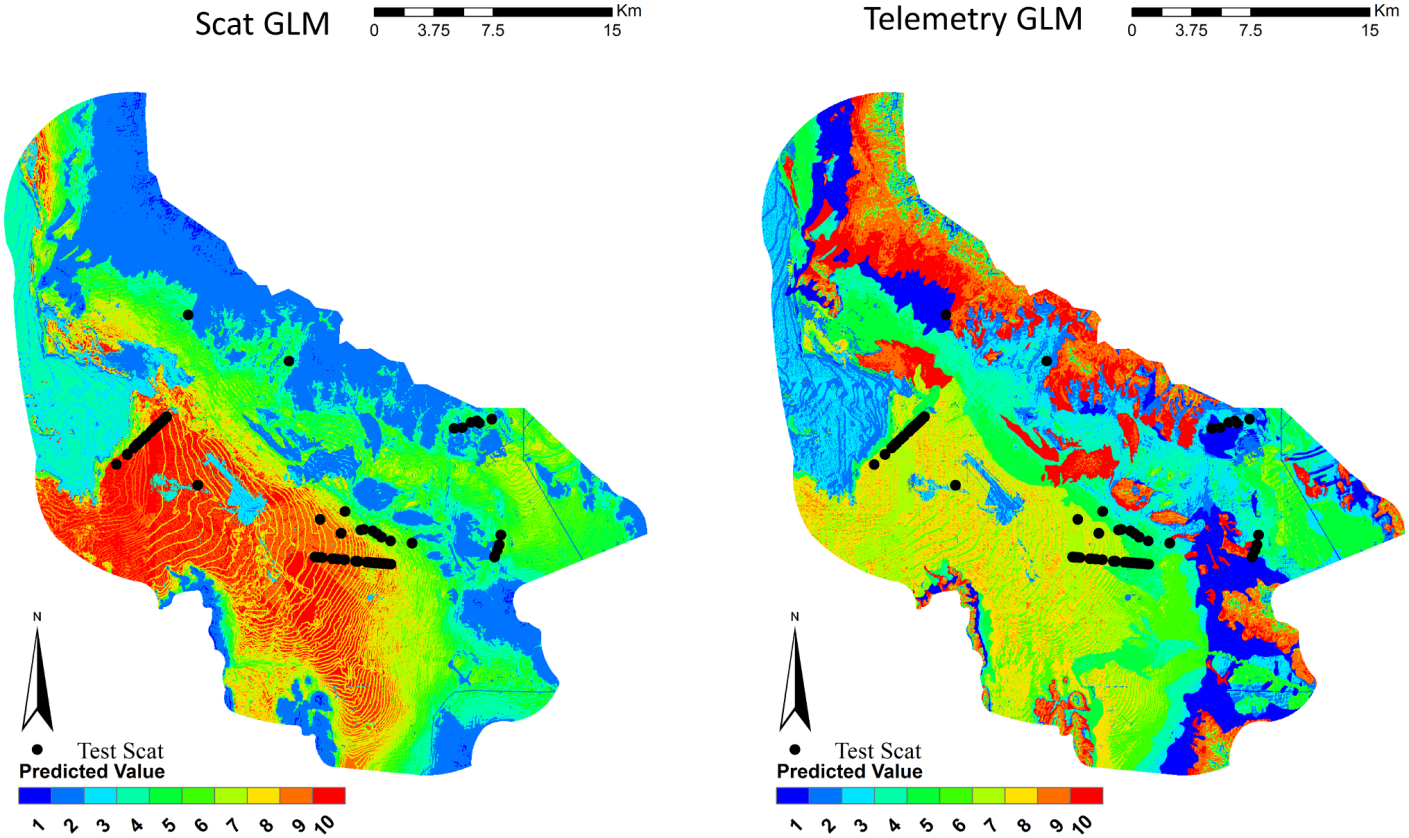
Species distribution models for kit foxes as generated by fixed-effect GLM using scat deposition transects and telemetry data for kit foxes, Dugway Proving Ground, Utah. Kit fox habitat quality is shown in quantile ranked categories from 1 (low quality) to 10 (high quality). Locations of kit fox scats from the subsequent testing dataset are plotted.

**Fig 3:**
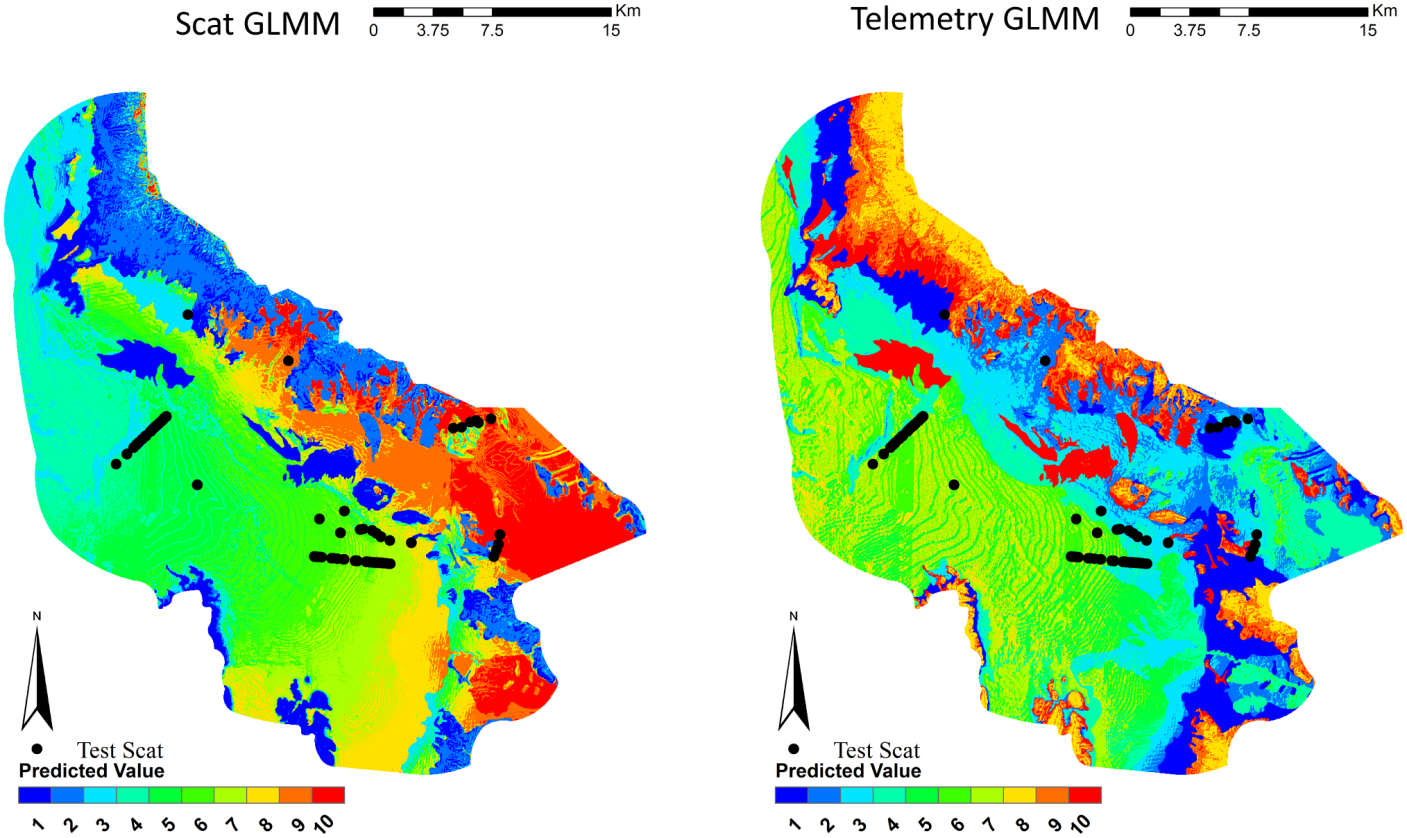
Species distribution models for kit foxes as generated by mixed-effect GLMM using scat deposition transects and telemetry data for kit foxes, Dugway Proving Ground, Utah. Kit fox habitat quality is shown in quantile ranked categories from 1 (low quality) to 10 (high quality). Locations of kit fox scats from the subsequent testing dataset are plotted.

**Fig 4:**
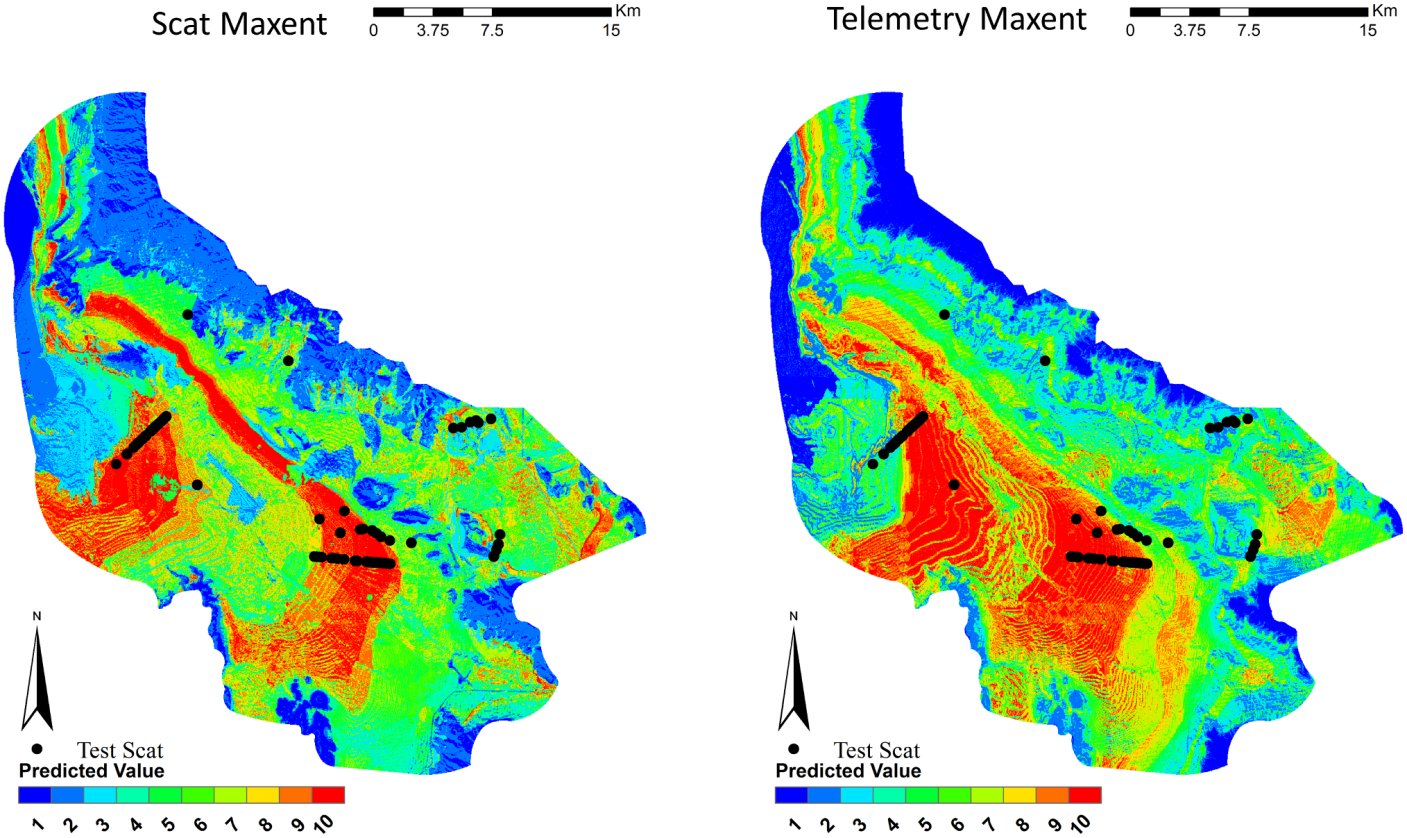
Species distribution models for kit foxes as generated by Maxent using scat deposition transects and telemetry data for kit foxes, Dugway Proving Ground, Utah. Kit fox habitat quality is shown in quantile ranked categories from 1 (low quality) to 10 (high quality). Locations of kit fox scats from the subsequent testing dataset are plotted.

**Fig 5:**
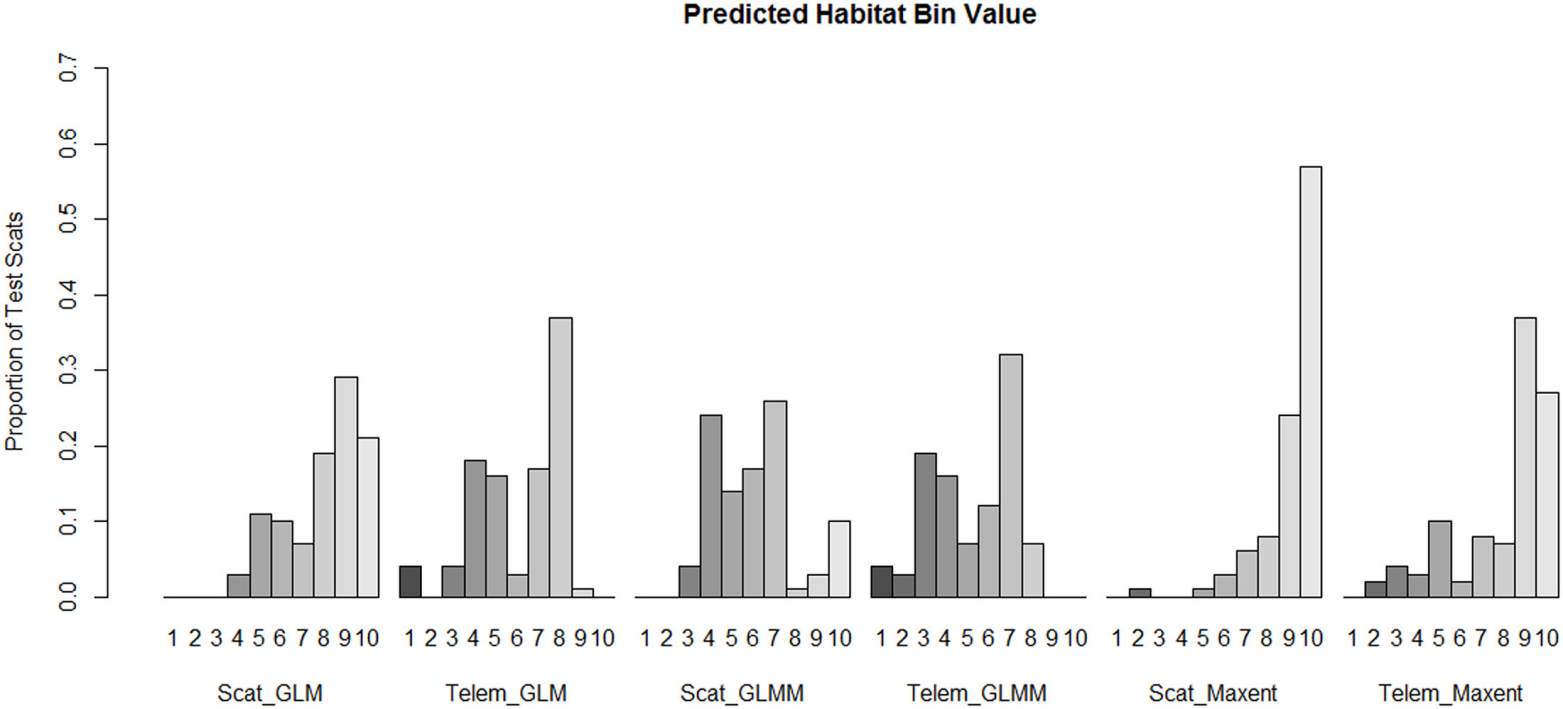
Proportion of test scat locations found in predicted habitat values from species distribution models, Dugway Proving Ground, Utah. Predicted values range from 1 –low quality habitat to 10—high quality habitat.

Species distribution maps were created for each model (Figs 2–4). Visual inspection of the scat GLM predicted that the low-lying flat portion of the study was of the highest habitat values. The telemetry GLM predicted this same area in mid to high habitat quality. It also selected the high, steep elevations as the top habitat quality. This appears to be a poor prediction, as kit foxes were not known to use such habitats and there were no “use” detections in that habitat. The scat GLMM concentrated most of the high quality in an area that was predominately flat at mid-elevation with most of the vegetation in this area consisting of invasive cheatgrass. The telemetry GLMM was similar to the telemetry GLM by selecting the low-lying flat portion of the study area as mid to high habitat quality and selected the high, steep elevations as the top habitat quality. Both of the Maxent models appeared to select the lowest-lying flat area as the highest quality. Across all models, when the test scats were overlaid on the distribution maps, very few scats occurred in the lowest predicted habitat values (Table 3, Fig 5). Both the GLM and Maxent models predicted higher quality habitats where the test scats occurred. The GLMM models selected mid-quality habitat values for where the majority of test scats occurred.

**Table 3:** Count of test scats from kit foxes found within predicted habitat quality from six different species distribution models, Dugway Proving Ground, Utah.

Predicted Habitat Value	Scat GLM	Telemetry GLM	Scat GLMM	Telemetry GLMM	Scat Maxent	Telemetry Maxent
1	0	4	0	4	0	0
2	0	0	0	3	1	2
3	0	4	4	17	0	4
4	3	16	22	14	0	3
5	10	14	13	6	1	9
6	9	3	15	11	3	2
7	6	15	23	29	5	7
8	17	33	1	6	7	6
9	26	1	3	0	22	33
10	19	0	9	0	51	24

To evaluate if a noninvasive method performed similarly to the invasive method in predicting the distribution of kit foxes, we compared the frequency of the test scats (Table 3) amongst the three noninvasive distribution maps generated from the scat transects versus the distribution maps produced from the radio-telemetry locations (invasive method). All noninvasive methods produced significantly different test scat bin locations then the invasive methods (GLM: χ^2^ = 70.00, 9 *df*, *P* < 0.001; GLMM: χ^2^ = 50.61, 2 *df*, *P* = <0.001; Maxent: χ^2^ = 7.63, 2 *df*, *P* = 0.022) indicating that the distribution models generated from the scat deposition surveys were different than the models generated from the telemetry dataset.

## Discussion

Our study demonstrated the importance of comparing multiple model types (e.g., fixed effects, mixed effects, Maxent) for development of resource selection functions used to predict a species distribution, and evaluating the importance of environmental variables on species distribution. All models we examined did show a large effect of elevation on kit fox presence. But had we only used the fixed-effect models, it would appear that elevation, slope, and vegetation height had nearly equal influence on kit fox occurrence. The AUC scores and inspection of testing data suggested the Maxent model appeared to perform the best, with elevation as a primary contributor, with smaller effects from slope, vegetation height, and soil type.

We applied a noninvasive survey technique that has been shown to be one of the best methods for detecting occurrence of carnivores [25, 29, 60, 61], and was relatively low in cost, resilient to weather, had low labor requirements, and posed no risk to the study animals (i.e., a noninvasive technique) [29, 60]. For many carnivore species, obtaining sufficient data on species presence can be difficult and expensive, limiting the development of informative models of species distribution. However, if we consider the dataset from the invasive sampling method (i.e., radio-telemetry locations) to be the most accurate depiction of resource selection and therefore the most useful to predict kit fox distribution on the study area, when we compared these maps the species distribution maps created from the noninvasive sampling (i.e., scat transects) were significantly different in terms of where the test scats should have occurred. Thus, while scat deposition transects may be useful for monitoring kit fox abundance [60], they did not appear to be appropriate for determining resource selection. Using scat transects to predict species distribution would require a more robust sampling design than we employed in our study. Our scat transects were constrained to roads, and therefore biased to lower elevations and flatter terrain. Increasing sampling to random transects that cover all types of habitats and topography would likely have given us a different result and conclusion.

The models built using the telemetry locations to produce the distribution maps should be more robust and reflective of kit fox spatial movements and resource selection, as the model inputs (e.g., presence data) used were “true” animal locations created from ~4,500 telemetry locations, and was not restricted or biased by collecting scat “use” data along roadways. Most roadways are placed on the landscape and constructed by engineers and therefore are at lower elevations and on flatter topography. In contrast, the telemetry dataset was dictated by movements of the kit foxes themselves, not road engineers.

The mixed-effects models included a random intercept to help account for variables of importance that were not included in the model, unbalanced sampling, variance among regions, and time [41, 43]. Additionally, the random effect allowed for extrapolation of the model [41] into new areas, allowing for application of the model outside the area studied. Unfortunately, these models appeared to over value mid-elevations and did not provide much insight into where kit foxes should occur. When plotting the test dataset it became apparent that these models predicted fox presence in lower quality habitats. The primary difference in these models was they selected a slightly higher elevation than both the Maxent model and the fixed-effects models, as the most highly valued habitat.

One study suggested vegetative cover was the most important environmental variable for modeling kit fox habitat using their available data [19]. They noted soil type was most likely of high importance and possibly other environmental variables. Today, with the wide variety and easy access to extensive spatial data, more complex models can be constructed. In this study, our results suggested that in this ecosystem, elevation was highly important to kit fox space use, followed by slope, vegetation height, and soil type. Elevation’s strong contribution to the models may be from its relationship to other environmental variables in this system that was not included in this models including: ruggedness, rainfall, water availability, and even climatic factors. Similar to other results [8], in our study area kit foxes appeared to occur in areas with elevations <1600 m.

Kit foxes were found to occur more frequently in areas with taller vegetation height, although this was only a moderate contribution to the models. It was difficult to determine how vegetation height was influencing kit fox presence. Vegetation height was related to many variables that may affect kit fox presence such as vegetation type, which may also influence the level of prey available in those areas. Vegetation height was also related to the amount of visibility for detection of prey and avoidance of predators, but this was likely confounded by vegetation density. More inference may be made by including those variables to help determine how vegetation influenced kit fox occurrence.

The response to soil type appeared to be driven by the importance of den sites to kit foxes. Kit foxes rarely occur in areas with large rocky soils which would be difficult for den excavation. Den sites are considered to be important to kit foxes as they “provide shelter from temperature extreme, moist microclimate, escape from predators, and a place to rear young” [15] and are a critical part of the survival strategy of kit foxes [19]. Therefore the proper conditions (i.e., denning substrate and surrounding habitat) may be required for kit foxes. Although kit foxes are highly mobile and capable of traveling away from denning areas to forage, they still tend to occur on soils where dens are easily dug. This may suggest kit fox stay within ‘den friendly’ soils because of the use of extensive den systems for refuge from predation [15, 62, 63].

The effect of slope was as expected from prior knowledge that kit foxes occur on mostly flat terrain [11, 17]. The contribution of slope maybe greater in more rugged areas, but the study area was mostly flat reducing the importance of slope in the models. Aspect was included due to reports that kit foxes often select dens with southern den entrances [16], which were hypothesized to provide thermal and microclimate advantages [15]. The low model contribution of aspect, which showed higher use of southern facing aspects, may be related to foxes using southern aspects for increased warmth from the sun.

For future studies, random placement of scat deposition transects on the landscape that are not constrained to roads may increase the utility of scat transects for determining models of a species distribution. However, detection probabilities of kit fox scats on transects that are located through the native vegetation would likely decline and the efficacy of random transects would have to be considered. Using scat-detection dogs may remedy the issue of finding scats as their detection abilities far exceed humans when locating scats along random transects placed through native vegetation [22, 64]. A study in Brazil determined the probability of occurrence of five mammalian species using scat-detection dogs surveying quadrats of different size and concluded that scat-detection dogs were an effective survey tool for rare species in the Brazilian Cerrado [65]. Future studies applying noninvasive scat sampling will need to consider a more robust random sampling design should their research objectives include determining kit fox distribution.

## Supporting Information

S1 Data Data files and layers for scat deposition transects, radio-telemetry locations, test scats, and environmental variables (aspect, elevation, slope, soils, vegetation height). (ZIP)
